# CRISPR/Cas9-mediated genomic insertion of functional genes into *Lactiplantibacillus plantarum* WCFS1

**DOI:** 10.1128/spectrum.02025-24

**Published:** 2025-01-16

**Authors:** Kamilla Wiull, Lisa K. Haugen, Vincent G. H. Eijsink, Geir Mathiesen

**Affiliations:** 1 Faculty of Chemistry, Biotechnology and Food Science, NMBU - Norwegian University of Life Sciences, Ås, Norway; University of Manitoba, Winnipeg, Canada

**Keywords:** genome editing, gene insertion, *Lactiplantibacillus plantarum*, CRISPR, surface display

## Abstract

*Lactiplantibacillus plantarum,* a natural inhabitant of the human body, is a promising candidate vehicle for vaccine delivery. An obstacle in developing bacterial delivery vehicles is generating a production strain that lacks antibiotic resistance genes and contains minimal foreign DNA. To deal with this obstacle, we have constructed a finetuned, inducible two-plasmid CRISPR/Cas9-system for chromosomal gene insertion in *L. plantarum*. The knock-in plasmid was designed with a cassette-like structure to simplify the insertion of target DNA and streamline the CRISPR/Cas9 genome editing, bringing it one step closer to becoming a routine procedure. We demonstrate that the system enables efficient insertion of expression cassettes for both inducible and constitutive production of a fluorescent reporter protein, mCherry, and for inducible production of the receptor-binding domain (RBD) of the SARS-CoV-2 virus. Two variants of RBD were successfully expressed, one directed to the cytoplasm and one directed to the cell surface. All the knock-in strains produced the target protein, although with lower yields than strains with plasmid-encoded expression.

**IMPORTANCE:**

Genetic engineering of lactic acid bacteria, such as *Lactiplantibacillus plantarum,* has proven to be difficult. This study presents an inducible two-plasmid CRISPR/Cas9-system for inserting genes into the chromosome of *Lactiplantibacillus plantarum*. Our system successfully knock-in four expression cassettes varying in length from ~800–1,300 bp with high efficiency and insert an expression cassette encoding a SARS-CoV-2 antigen receptor-binding domain (RBD) with an anchor mediating surface display, which has not been achieved previously using CRISPR/Cas9. We demonstrate the production of the insertion genes. Importantly, the plasmid carrying the SgRNA, Cas9, and homology-directed repair template is designed for easy component exchange. These plasmids represent valuable contributions to the field as they could facilitate rapid CRISPR/Cas9 engineering of *L. plantarum* strains.

## INTRODUCTION

*L. plantarum*, a lactic acid bacterium (LAB), emerges as a promising candidate for biotechnological engineering, particularly for delivery of medicinal proteins. LAB-based delivery vehicles have shown potential against various diseases and medical conditions (1–4). An important obstacle in this development is the need to construct strains that display stable protein production, while not carrying transferable antibiotic resistance genes or large amounts of foreign DNA. The need for antibiotic resistance genes can be circumvented by using hosts that lack an essential gene, allowing the host to grow only when the medium is supplemented with the gene product or when the host is transformed with a plasmid containing the missing essential gene (5–7). For example, T. T. Nguyen *et al*. (7) constructed a food grade expression system for *L. plantarum*, using the essential alanine racemase gene (*alr*) as a selection marker. The constructed food-grade plasmids, which are derivatives of the pSIP-plasmids for inducible gene expression in lactobacilli (8, 9), have been used for successful production of various enzymes in *L. plantarum* (10–12). However, these systems still yield production strains with considerable amounts of foreign (plasmid) DNA.

Over the years, several methods for knocking-in genes in lactobacilli have been developed (13). The *lox*P/Cre system is a vastly used tool for knock-in and knock-out of genes in *L. plantarum* (14–19). The system relies on the Cre recombinase and 34-bp *lox*P restriction sites. The need for *lox*P sites entails a main limitation of this system as the motif is not found abundantly in the genome. Often, the *lox*P sites are inserted into the genome together with an antibiotic resistance gene, which can be laborious to counter-select after successful genome editing. Furthermore, editing with the *lox*P/Cre system leaves a scar in the genome.

About one decade ago, the use of Clustered Regularly Interspaced Short Palindromic Repeats (CRISPR) was proposed as a new tool for scarless genomic engineering (20, 21). Upon discovery of the CRISPR tool, there was initial optimism that it would enable rapid gene modifications in all living organisms. As opposed to the 34-bp *lox*P sites required for *lox*P/Cre editing, the Cas9 enzyme recognizes a motif of only 3 bp: the 5′-NGG-3′ protospacer adjacent motif (PAM). However, for genomic engineering of bacteria, the system has proven more challenging than first thought. The cytotoxicity of the required heterologous Cas9 enzyme, most often derived from *Streptococcus pyogenes* (SpCas9 (13)), and lack of efficient repair systems may explain the experienced difficulties (22).

In *Lactocaseibacillus casei,* a mutated SpCas9 called nickase or SpCas9^D10A^ exhibited higher editing efficiency and lower cytotoxicity compared to wild-type SpCas9 (23). A downside of nickase-mediated gene knock-out or knock-in is the generation of both pure and mixed-genotype colonies. After performing experiments to optimize single-stranded break induced by SpCas9^D10A^, X. Song *et al*. (23) obtained a knock-out efficiency of 35% with SpCas9^D10A^, which included mixed and pure genotype colonies. The nickase system enabled chromosomal insertion (knock-in) of GFP, with an efficacy of 33%. Very recently, F. Li *et al*. (24) performed SpCas9^D10A^-mediated knock-in of PEDV1, an antigen isolated from porcine epidemic diarrhea virus (PEDV), into the thymidylate synthase (*thyA*) gene of *Lacticaseibacillus paracasei*. The authors showed that the engineered strain induced mucosal, humoral, and cellular immunity in mice after oral administration of bacteria harboring PEDV1 in the cytoplasm.

R. T. Leenay *et al*. (25) conducted research on *L. plantarum* and showed great variability in SpCas9-mediated point-mutation insertion and gene knock-out among three different *L. plantarum* strains, which highlights the importance of developing strain-specific CRISPR/Cas9-systems. D. Zhou *et al*. (26) successfully knocked-in promoter 3 a in front of a chromosomal glutamine–fructose-6-phosphate transaminase in *L. plantarum* WCFS1 using inducible expression of SpCas9. Notably, these authors used a two-step recombineering method, relying on the *lox*P/Cre system in combination with the CRISPR/Cas9-system, because their CRISPR/Cas9-system alone failed to generate knock-ins. H. Huang *et al*. (27) demonstrated CRISPR/Cas9-mediated insertion of a pyruvate decarboxylase (*pdc*) gene in *L. plantarum* WCFS1, through gene exchange between the 1,892-bp *pdc* gene and the 963-bp *lp_0537* gene. They showed high insertion efficacies of 35%–38%, but gene expression from the knock-in strains was not verified. In this case, a previously characterized recombinase operon from *L. plantarum* WCFS1 (18) was overexpressed to strengthen the homologous directed repair (HDR) mechanism that is needed to enable gene insertion. Despite these examples of successful CRISPR/Cas9-mediated genome engineering in lactobacilli, so far only one gene has successfully been inserted into the genome of *L. plantarum* WCFS1 using CRISPR/Cas9 only, and the efficiency of insertion was rather low.

To improve the CRISPR/Cas9 system for gene insertion into the genome of *L. plantarum* WCFS1, we have developed a gene knock-in system comprising two plasmids consecutively transformed into the bacterium. Notably, in contrast to other CRISPR/Cas9-mediated insertions in LAB, we did not remove genetic content from the genome through gene exchange as we inserted our expression cassettes into a short, noncoding region. The system was constructed to facilitate easy exchange of key components, such as the homologous arms that co-determine the site of insertion or the knock-in gene. Furthermore, we finetuned the CRISPR/Cas9-system by controlling expression levels of Cas9 using the inducible P*_sppA_* promoter and by assessing the effect of different levels of recombinase activity on recombination efficiency. This finetuning of the CRISPR/Cas9-system allowed us to successfully knock-in four genes into *L. plantarum* WCFS1, three of which were under control of an inducible promoter: mCherry and two forms of the receptor-binding domain (RBD) from the SARS-CoV-2 virus, one directed to the cytoplasm and one directed to the surface through a lipoprotein anchor. The fourth knock-in was constitutively expressed mCherry. Gene fragments of ~800–1,300 bp were knocked-in, and the expression was assessed by fluorescence measurements, Western blotting, and flow cytometry.

## MATERIALS AND METHODS

### Bacterial strains and growth conditions

All plasmids and bacterial strains used in this study are listed in Table 1. For plasmids carrying the rep256 replicon, *Escherichia coli* TOP10 was used as the subcloning host, while *Lactococcus lactis* IL403 was used as the subcloning host for plasmids carrying the repSh71 replicon (*L. lactis* was preferred over *L. plantarum* WCFS1 because of higher transformation efficiencies and easier purification of sufficient amounts of plasmid DNA). Chemically competent *E. coli* TOP10 (Invitrogen, Waltham, MA) was transformed following the manufacturer’s protocol and cultivated in Brain–Heart Infusion (BHI) medium (Oxoid Ltd., Carlsbad, CA) at 37°C with shaking (220 rpm). *L. lactis* was made electrocompetent and transformed as previously described (28) and cultivated in M17 (Oxoid) medium supplemented with 0.5% glucose (GM17) at 30°C without shaking. *L. plantarum* was made electrocompetent and transformed as previously described (29) and cultivated in Man, Rogosa, and Sharpe (MRS) broth (Oxoid) at 37°C without shaking. Solid BHI, MRS, and GM17 media were prepared by addition of 1.5% (wt/vol) agar. Chloramphenicol (Cm) was added to a final concentration of 34 µg/mL or 10 µg/mL for *E. coli* or LAB, respectively. Erythromycin (Ery) was added to a final concentration of 200 µg/mL or 10 µg/mL for *E. coli* or LAB, respectively.

**TABLE 1:** Strains and plasmids used in this study

	Description	Reference
Strain		
*E. coli* TOP10	Subcloning host	Invitrogen
*L. lactis* Il403	Subcloning host	(30)
*L. plantarum* WCFS1	Host strain	(31)
P*_sppA_-*mCherry	*L. plantarum* WCFS1 harboring pSIP403_mCherry (inducible promoter)	(4)
Lp::P*_sppA_-*mCherry	*L. plantarum* WCFS1 with knock-in of the P*_sppA_-mCherry* expression cassette, harboring pEV	This study
P*_slpA_*-mCherry	*L. plantarum* WCFS1 harboring pSIP403_SlpA_mCherry (constitutive promoter)	(4)
Lp::P*_slpA_*-mCherry	*L. plantarum* WCFS1 with knock-in of the P*_slpA_-mCherry* expression cassette	This study
P*_sppA_*-RBD	*L. plantarum* WCFS1 harboring pSIP_RBD	This study
Lp::P*_sppA_*-RBD	*L. plantarum* WCFS1 with knock-in of the P*_sppA_-RBD-DC* expression cassette, harboring pEV	This study
P*_sppA_*-1261-RBD	*L. plantarum* WCFS1 harboring pSIP_1261-RBD	This study
Lp::P*_sppA_*-1261-RBD	*L. plantarum* WCFS1 with knock-in of the P*_sppA_-1261-RBD-DC* expression cassette, harboring pEV	This study
Plasmid		
pSgRNA-acm2	Cm^r^, 256_rep_, P_3_:sgRNA-2645	(32)
pSgRNA-KI	Cm^r^, 256_rep_, P_3_:sgRNA-KI	This study
pSgRNA-KI_Cas9	Cm^r^, 256_rep_, P_3_:sgRNA-KI, P*_sppA_*:*cas9*	This study
pSIP411	pSIP-expression vector, Ery^r^, Sh71_rep_, P*_sppA_*:*gusA*	(9)
pCas	Kan^r^, repA101ts, encodes Cas9 from *Sreptococcus pyogenes*	Addgene
pSIP403_Cas9	pSIP-expression vector, Ery^r^, 256_rep_, P*_sppA_*:*cas9*. *cas9* from *S. pyogenes*	This study
pSIPSh71_LpRec	pSIP-expression vector, Ery^r^, 256_rep_, P*_sppIP_*:*sppK-sppR,* P*_sppA_*:*lp_0640–0642, lp_0640–0642* from *L. plantarum* WCFS1.	This study
pSIP403_mCherry	pSIP-expression vector, Ery^r^, 256_rep_, P*_sppA_*:*mCherry*	(4)
pSIP403_SlpA_mCherry	pSIP-expression vector, Ery^r^, 256_rep_, P*_slpA_*:*mCherry*	(4)
pLp_1261AgE6-DC	pSIP-expression vector, Ery^r^, 256_rep_, P*_SppA_*:*1261-AgE6*	(33)
pUC57_DC_NTD_RBD	Amp^r^, rep(pMB1), contains the antigens NTD and RBD from SARS-CoV-2 fused to a DC-binding peptide (DC).	GenScript
pSIP_RBD	pSIP-expression vector, Ery^r^, 256_rep_, P*_sppA_*:*RBD-DC*.	This study
pSIP_1261-RBD	pSIP-expression vector, Ery^r^, 256_rep_, P*_sppA_*:*1261-RBD-DC*. RBD is translationally fused to a lipoprotein anchor, 1261, derived from *lp_1261*.	This study
pEV	pSIP-expression vector, Ery^r^, 256_rep_, “empty vector” control plasmid, P*_sppIP_*:*sppK-sppR*	(34)
pCRISPR-P*_sppA_*-mCherry	Cm^r^, 256_rep_, P*_sppA_*:*cas9*, P_3_:sgRNA-KI, P*_sppA_*-mCherry	This study. Deposited to Addgene
pCRISPR-P*_slpA_*-mCherry	Cm^r^, 256_rep_, P*_sppA_*:*cas9*, P_3_:sgRNA-KI, P*_slpA_*-mCherry	This study
pCRISPR-P*_sppA_*-RBD	Cm^r^, 256_rep_, P*_sppA_*:*cas9*, P_3_:sgRNA-KI, P*_sppA_-RBD-DC*.	This study
pCRISPR-P*_sppA_*-1261-RBD	Cm^r^, 256_rep_, P*_sppA_*:*cas9*, P_3_:sgRNA-KI, P*_sppA_-1261-RBD-DC*. RBD is translationally fused to a lipoprotein anchor (1261).	This study

### Plasmid construction

To construct pSIPSh71_LpRec with inducible overexpression of the recombinase operon (*lp0640-42*) from *L. plantarum* WCFS1, *lp0640-42* was first amplified using genomic DNA of *L. plantarum* WCFS1 as the template, with primers SppA-LpRec_F and SgRNA-LpRec_R (Table S1). The inducible P*_sppA_* promoter was amplified using the primer pair SgRNA-LpRec_F/SppA-LpRec_R with pSIP403_mCherry as the template (Table S1). The two fragments (with 12 overlapping base pairs) were subsequently mixed and fused together in an overlap extension-PCR using the outer primers SgRNA-LpRec_F and SgRNA-LpRec_R to fuse the recombinase operon to P*_sppA_*. Subsequently, both the fused PCR product and the pSIP411 vector were digested with *Age*I-HF/*Xma*I (both New England Biolabs (NEB), Ipswich, MA). The digested fragments were ligated using electroligase (NEB) according to the manufacturer’s protocol, followed by transformation of the ligated DNA into electrocompetent *L. lactis* to yield the plasmid pSIPSh71_LpRec (Table 1).

The sequence of a gene fragment encoding the RBD from the spike protein of SARS-CoV-2, fused to a DC-binding peptide (35), was optimized for *L. plantarum* and purchased from GenScript (Piscataway, NJ). The RBD fragment was amplified from the purchased pUC57_DC_NTD_RBD plasmid with primer pairs RBD_F/RBD_R and 1261_RBD_F/1261_RBD_R, and InFusion (Takara Bio, Kusatu, Japan) cloned, according to the manufacturer’s protocol, into *Nde*I/*Hind*III digested pLp1261AgE6-DC (Table 1) and *Sal*I/*Hind*III digested pLp1261AgE6-DC to obtain pSIP_RBD and pSIP_1261-RBD (Table 1).

The genomic site chosen for CRISPR/Cas9-mediated knock-in was a noncoding region between *lp_2071* and *lp_2074*, which are transcribed in opposite directions. The *lp_2071* gene is followed by a terminator, which prevents read-through from *lp_2071* into the inserted gene. The inserted expression cassettes with the P*_sppA_* promoter contain an additional terminator directly upstream of the promoter. The sgRNA used for guiding Cas9 to the chosen genomic site is named sgRNA-KI. The pCRISPR-plasmids, containing all necessary components (sgRNA, Cas9, and the HDR-template), were constructed in three steps. In the first step, sgRNA-acm2 in the previously described pSgRNA-acm2 plasmid (32) was replaced with sgRNA-KI by using inverse PCR. In the inverse PCR, the forward primer (SgRNA_KI1_F) binds downstream of sgRNA-acm2 in pSgRNA-acm2 with a 5’ tail containing the 20-nt sgRNA-KI sequence (5′-ATAAACGACTTCGGTGGAAT-3′). The phosphorylated reverse primer (Phospho-sgRNA_R) binds directly upstream of the guide RNA (Table S1). PCR was conducted using Q5 High-Fidelity DNA polymerase (NEB). The 3,150-bp PCR-product was isolated using the NucleoSpin Gel and PCR Clean-up kit (Macherey-Nagel). The purified PCR-product was digested with *Dpn*I (NEB) for 20 minutes at 37°C to remove the PCR template, followed by a second clean-up with the NucleoSpin Gel and PCR Clean-up kit. The purified PCR-fragment was then subjected to self-ligation using the NEB Quick ligase, according to the manufacturer’s protocol, and transformed to chemically competent *E. coli* TOP10 cells, according to the manufacturer’s instructions, to obtain pSgRNA-KI. Correct pSgRNA-KI clones were confirmed by DNA sequencing.

In the second step, Cas9 was amplified from pCas9 (Table 1) with primer pair Cas9NcoI_F/pCasR, and InFusion cloned into *Nco*I/*Xho*I digested pSIP403, downstream of the inducible promoter P*_sppA_*, to obtain pSIP403_Cas9. P*_sppA_*-Cas9 was amplified from pSIP403_Cas9 with primers SgRNAKI-1-HA_cas9_F and Sg-Cas9_R, and InFusion cloned into *Xho*I/*Xma*I digested pSgRNAKI1, to obtain pSgRNA-KI_Cas9.

**Fig 1:**
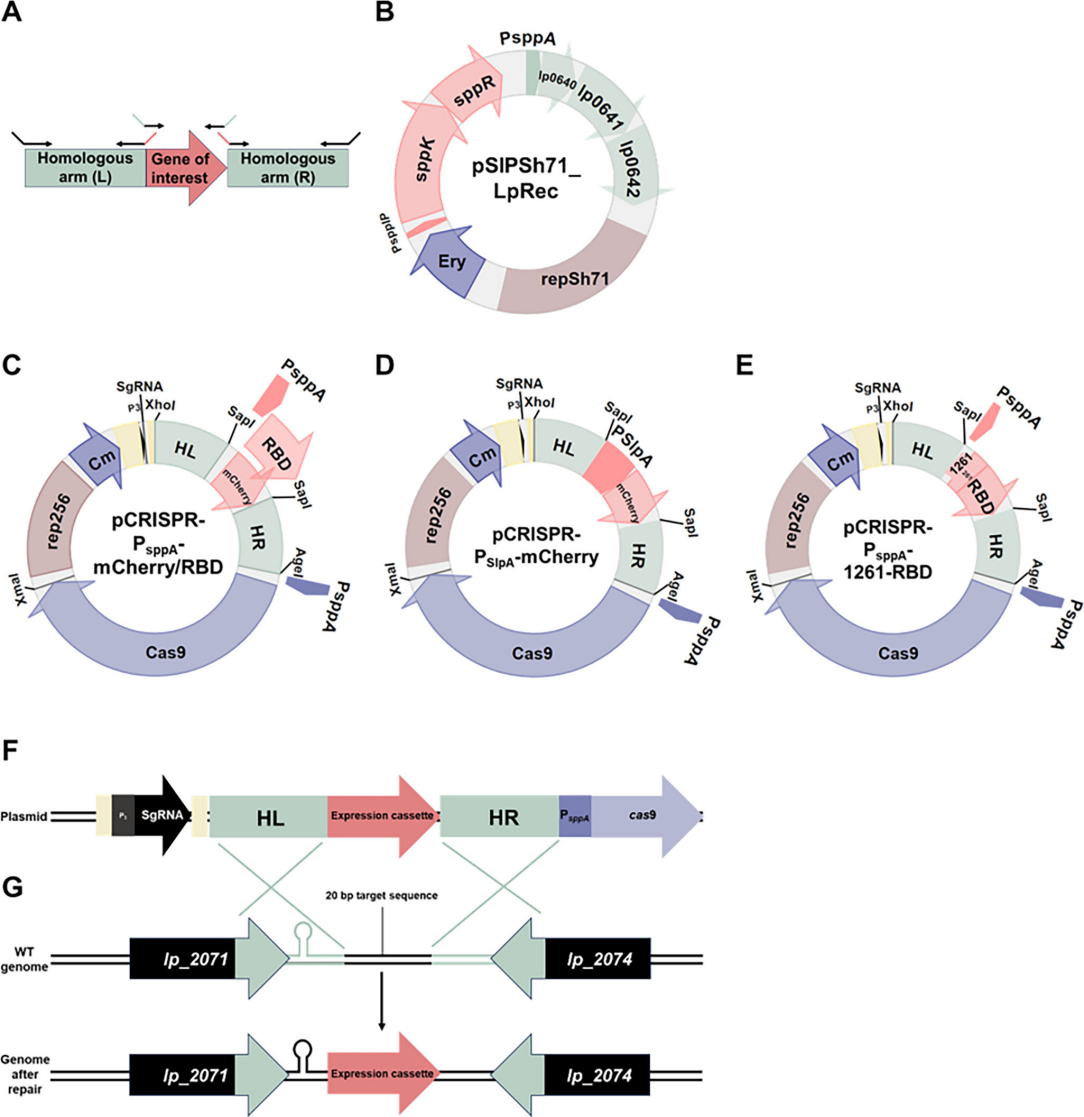
A schematic overview of the plasmids used for gene knock-in. Panel (**A**) shows the design of the homology-directed repair (HDR)-template, which was generated using overlap PCR. The 1-kb homologous arms (in green) upstream (**L**) and downstream (**R**) of the gene of interest (in red) were amplified with primers containing overlapping tails (illustrated with green and red colors). After amplifying the three individual fragments, the fragments were combined into one fragment through extension PCR in one PCR, using the outer primers only (shown as all black primers). Panel (**B**) shows the pSIPSh71_LpRec plasmid for recombinase expression, while panels (**C–E**) give an overview of the pCRISPR-plasmids (note that panel C shows two plasmids in one picture). Replication of pSIPSh71_LpRec and the pCRISPR plasmids is controlled by different but compatible replicons, repSh71 in pSIPSh71_LpRec and rep256 in the pCRISPR plasmids. Furthermore, while the pSIPSh71_LpRec plasmid carries an erythromycin (Ery) resistance gene, the pCRISPR plasmids carry a chloramphenicol (Cm) resistance gene. The homologous arms (HL and HR) flank the different promoters and genes of interest in all pCRISPR plasmids. Terminators located upstream the P_3_ promoter (black arrow) and downstream of the sgRNA (shown as a tick mark) are shown in yellow. The indicated restriction sites (*Xho*I, *Sap*I, *Age*I, and *Xma*I) contribute to a cassette-like structure of the plasmids, facilitating exchange of each component. Panel (**F**) shows a schematic overview of a section of the plasmids in C–E, where the different expression cassettes shown in C–E are denoted “expression cassette” for simplicity. Panel (**G**) shows the parts of the plasmid and genome where homology-directed repair occurs, as well as the resulting edited genome with knock-in of a heterologous expression cassette. The terminator located downstream of the *lp_2071* gene is shown as a lollipop..

In the third step, first, a DNA fragment representing the gene intended for knock-in was flanked with 1-kb long fragments identical to the *L. plantarum* genome on each side of the Cas9 target site, using overlap-extension PCR (Fig. 1A). The homologous fragments serve as the repair template necessary for homologous directed repair (HDR). The HDR template was constructed by amplifying the homologous arms, which were named homology left (HL) and homology right (HR), using primer pairs pSgKI1-HL_XhoI_F/HL_2071–2074_SapI_rev and H-2071–2074-R_fwd/pSgKI1-HR_AgeI_R, respectively. The NEBuilder Assembly web-tool (NEB) was used to construct primers with overhangs complementary to the terminuses of the knock-in expression cassette, resulting in primers with 7–27 bp overhangs. Second, a knock-in expression cassette, P*_sppA_*-RBD, was amplified with primers containing tails (20–21 bp) homologous to the terminus of either the HL (the forward primer) or the HR (the reverse primer), with primer pair Psppa-RBD_fwd/KI1_HR_RBD_R (Fig. 1A). After amplifying the arms and the knock-in expression cassette in three separate PCRs, the three purified PCR fragments were fused in a second PCR, using outer primers pSgKI1-HL_XhoI_F and pSgKI1-HR_AgeI_R (depicted as black arrows in Fig. 1A). The resulting 2,844-bp fragment HL-P*_sppA_*-RBD-HR (HA-RBD) was subsequently cloned into *Xho*I*Age*I digested pSgRNA-KI_Cas9 (Table 1) using the InFusion cloning kit, yielding pCRISPR-P*_sppA_*-RBD.

For the construction of the three additional pCRISPR plasmids (Fig. 1C and E), the knock-in expression cassettes were amplified from pSIP403_mCherry, pSIP403_SlpA_mCherry, and pSIP_1261-RBD with primer pairs PsppA-RBD_fwd/KI1_HR_mCherry_R, HL_slpA-RBD_fwd/ KI1_HR_mCherry_R, and PsppA-RBD_fwd/KI1_HR_RBD_R, respectively, and InFusion cloned into *Sap*I digested pCRISPR-P*_sppA_*-RBD to obtain pCRISPR-P*_sppA_*-mCherry, pCRISPR-P*_slpA_*-mCherry, and pCRISPR-P*_sppA_*-1261-RBD. All the pCRISPR plasmids were sequenced prior to further usage. The pCRISPR-P*_sppA_*-mCherry plasmid has been deposited to Addgene.

### Transformation of *L. plantarum* to obtain knock-in colonies

*L. plantarum* WCFS1 harboring pSIPSh71_LpRec were made competent as mentioned previously and induced as described in (27). In short, at OD_600_ = 0.3 ± .03, the cells were induced with SppIP to a final concentration of either 0.25 ng/mL or 25 ng/mL SppIP (36). A third culture was left uninduced. The competent cells were harvested after approximately 2 hours of growth (at OD_600_ = 0.7 ± .07 as per the protocol for making competent cells). The competent cells were transformed with 5 µL pCRISPR plasmid and incubated for 2 hours in 950 µL MRSSM (MRS; 0.5 M sucrose; 0.1 M MgCl_2_). After the incubation, 100 µL of the transformation mixes was spread on MRS plates containing 10 µg/mL Ery and 10 µg/mL Cm. Colonies appeared after 2 days and were screened with PCR using the primer pair 2071–2074_SekF/2071–2074_SekR to detect knock-in colonies.

### Plasmid curing

The knock-in strains were first inoculated from glycerol stocks and incubated overnight in 10 mL MRS. The next day, 50 µL culture was streaked out with an inoculating loop on MRS agar dishes to obtain single colonies. Single colonies were transferred to individual wells in a 96-well plate, containing 200 µL MRS. After ~3 hours of incubation in the MRS medium, 10-µL portions of the culture were transferred to two new wells containing 190 µL MRS with 10 µg/mL Ery or 10 µg/mL Cm. Cultures that grew only in the MRS without antibiotics were further cultivated in 10 mL MRS to make glycerol stocks.

### Analysis of mCherry-fluorescence

The fluorescence of the mCherry knock-in strains was measured in microtiter plates using a Varioskan Lux Reader set at 37°C (ThermoFisher Scientific, Waltham, MA). Overnight cultures of the recombinant strains were diluted to OD_600_ 0.15 in prewarmed MRS and grown until OD_600_ ~0.30 ± .03. At this OD_600_, cells containing plasmids with an inducible promoter were induced with SppIP to a final concentration of 25 ng/mL SppIP. The cultures with constitutive expression of mCherry were not induced. Subsequently, 200 µL of the cultures was transferred to a sterile 96-well plate. The OD_600_ and fluorescence (Ex_587nm_; Em_620nm_) of the cultures were measured concomitantly during incubation for 24 hours at 37 ° C without shaking.

### Western blot analysis

Cultures were grown and induced (only for strains with inducible expression of RBD), as described in the previous section, and 10 mL culture was harvested 3 hours after induction by centrifugation (5,000 g, 10 minutes, 4°C). The cell pellets were washed once with 5 mL PBS (pH 7.5, 5,000 g, 10 minutes, 4°C) and resuspended in 1 mL PBS. The suspensions were transferred to FastPrep tubes containing 0.5 g acid-washed glass beads (Sigma-Aldrich, Saint-Louis, MO) and lysed in a FastPrep FP120 Cell Disrupter (MP Biomedicals, Santa Ana, CA) with three rounds of shaking at 6.5 m/s for 45 seconds. The samples were kept on ice for 5 minutes between each round. After cell lysis, the samples were centrifuged for 1 minute at 16,000 g at 4°C, and the cell-free crude lysates were transferred to new Eppendorf tubes. The samples were prepared for SDS-PAGE (Bio-Rad, Hercules, CA) by mixing 20-µL sample with 3 µL 10X NuPAGE Sample Reducing Agent and 7.5 µL 4X NuPAGE LDS Sample Buffer (both from Thermo Fisher Scientific), followed by incubation in a 100°C heating block for 10 minutes. The volumes loaded onto the gel were normalized based on the OD_600_ of the culture at harvesting. The gel was run for 30 minutes at 200 V. After the run, the proteins were transferred to a nitrocellulose membrane using an iBlot Dry Blot System (Invitrogen). Subsequently, the membrane was immunohybridized using the SNAP i.d. 2.0 protein detection system (Sigma-Aldrich). For the immunohybridization, the membrane was first blocked in 30 mL 4% (wt/vol) BSA in TTBS (0.01% Tween-20 (vol/vol), TBS pH 7.5). The rabbit polyclonal primary antibody SARS-CoV-2 Spike RBD (MBS2563840, MyBioSource, San Diego, CA) was diluted 1:2500 in 5 mL 4% BSA/TTBS and incubated with the membrane for 10 minutes. After the incubation, the membrane was washed three times with 30 mL TTBS. The HRP-Goat Anti-Rabbit secondary antibody (Invitrogen) was diluted 1:25000 in 5 mL 4% BSA/TTBS, and the membrane was incubated with the antibody for 10 minutes, followed by four washes with 30 mL TTBS. After the final wash, the membrane was placed in a plastic container and incubated for 5 minutes with SuperSignal West Pico PLUS Chemiluminescent substrate (ThermoFisher Scientific) according to the manufacturer’s protocol. Immunohybridization was visualized by imaging the membrane using an Azure c400 system (Azure biosystems, Dublin, CA).

### Flow cytometry analysis

From 10-mL cultures, grown and induced as described above, aliquots of 600 µL were harvested 3 hours after induction (5,000 g, 4 minutes, RT). The pellets were washed once with 600 µL PBS (pH 7.5) and subjected to lysozyme treatment to facilitate secondary antibody binding. To do so, the harvested bacteria were resuspended in a solution obtained by mixing 25 µL 100 mg/mL lysozyme with 225 µL PBS and incubated for 20 minutes at 37°C. After the lysozyme treatment, the cells were washed once with 600 µL PBS and resuspended in a mixture of 1 µL rabbit polyclonal primary antibody against SARS-CoV-2 Spike RBD (MyBioSource) in 50 µL PBS. The cell suspension was incubated at RT for 30 minutes and washed three times with 600 µL PBS (8,000 g, 2 minutes, RT). The cells were then resuspended in a mixture of 0.5 µL Goat anti-Rabbit IgG (H + L) Secondary antibody conjugated to PE (ThermoFisher Scientific) in 50 µL PBS and incubated for 30 minutes at 37°C in the dark. Subsequently, the bacteria were washed four times with 600 µL PBS (8,000 g, 2 minutes, RT) and resuspended in 1 mL PBS. The cell suspension was subjected to a 1:10 dilution in PBS in the flow cytometry sample tubes and analyzed using a MACSQuant analyzer (Miltenyi Biotec GmbH, Bergisch Gladbach, Germany). The instrument was set to register 30,000 events. Data were analyzed using the FlowJo software (BD Bioscience, Franklin Lakes, NJ).

## RESULTS

### Design of the CRISPR/Cas9-system for *L. plantarum* WCFS1

We constructed a set of two plasmids for CRISPR/Cas9-mediated gene insertion in *L. plantarum* WCFS1, as outlined in Fig. 1. To test the system, we attempted to knock-in two genes (RBD and mCherry) under the control of either an inducible or a constitutive promoter in a noncoding region (between *lp_2071* and *lp_2074*) of the *L. plantarum* WCFS1 genome. The length of the gene fragments ranged from ~800 to ~1,300 bp. In three cases, the protein of interest was targeted to the cytoplasm, while the fourth construct was designed to direct the RBD protein to the cell surface using a lipoprotein anchor. Cloning efficiency is significantly lower when the target gene is controlled by a constitutive promoter, such as P*_slpA_*, compared to a tightly controlled inducible promoter, such as P*_sppA_*. We speculate that this is due to the constitutive production of the heterologous protein since gene expression initiates simultaneously with the transformation of the expression vector. Therefore, we chose to include both types of promoters in the knock-in experiments. Insertion of the RBD antigen into the chromosome of *L. plantarum* is of interest, especially the surface-displayed version, as this could open up for development of novel vaccines against SARS-CoV-2.

In the two-plasmid CRISPR/Cas9 system (Fig. 1), one plasmid carries the recombinase operon (*lp0640, lp0641,* and *lp0642*) derived from *L. plantarum* WCFS1. The operon was placed under control of the strong, inducible pSIP promoter P*_sppA_* (8) (Fig. 1B) since it has been shown that overexpression of recombinases improves homology-directed repair (HDR) events (18). Initially, we attempted to make gene knock-ins without overexpressing *lp_0640–42*, which failed to yield recombinant colonies (data not shown). The second plasmid (Fig. 1C and E) harbors an HDR template (Fig. 1A) and encodes the two other components needed for a complete CRISPR/Cas9 HDR-system, the sgRNA sequence and a gene encoding Cas9. The HDR template (Fig. 1A) was constructed using overlap-extension PCR and contains 1-kb homologous arms (HL and HR) on both sides of the to-be knocked-in expression cassette. The expression of Cas9 is under control of the same inducible promoter (P*_sppA_*) as the recombinase operon. The P*_sppA_* have shown weak constitutive expression without induction, which is shown in (32) to be sufficient expression in terms of Cas9 experiments. We exploited this trait in our set-up as overexpression of heterologous Cas9 has been shown to be cytotoxic (22). The pCRISPR plasmids were designed with introduction of unique restriction sites for fast and easy exchange of the knock-in gene (*Sap*I restriction site), enabling generation of new knock-in strains within a time frame of 4 to 5 days. The CRISPR/Cas9 components of the plasmids in Fig. 1C and E are shown schematically in Fig. 1F, and the insertion of the expression cassettes into the targeted locus in the genome is indicated in Fig. 1G.

### Performance of the CRISPR/Cas9-system

**Fig 2:**
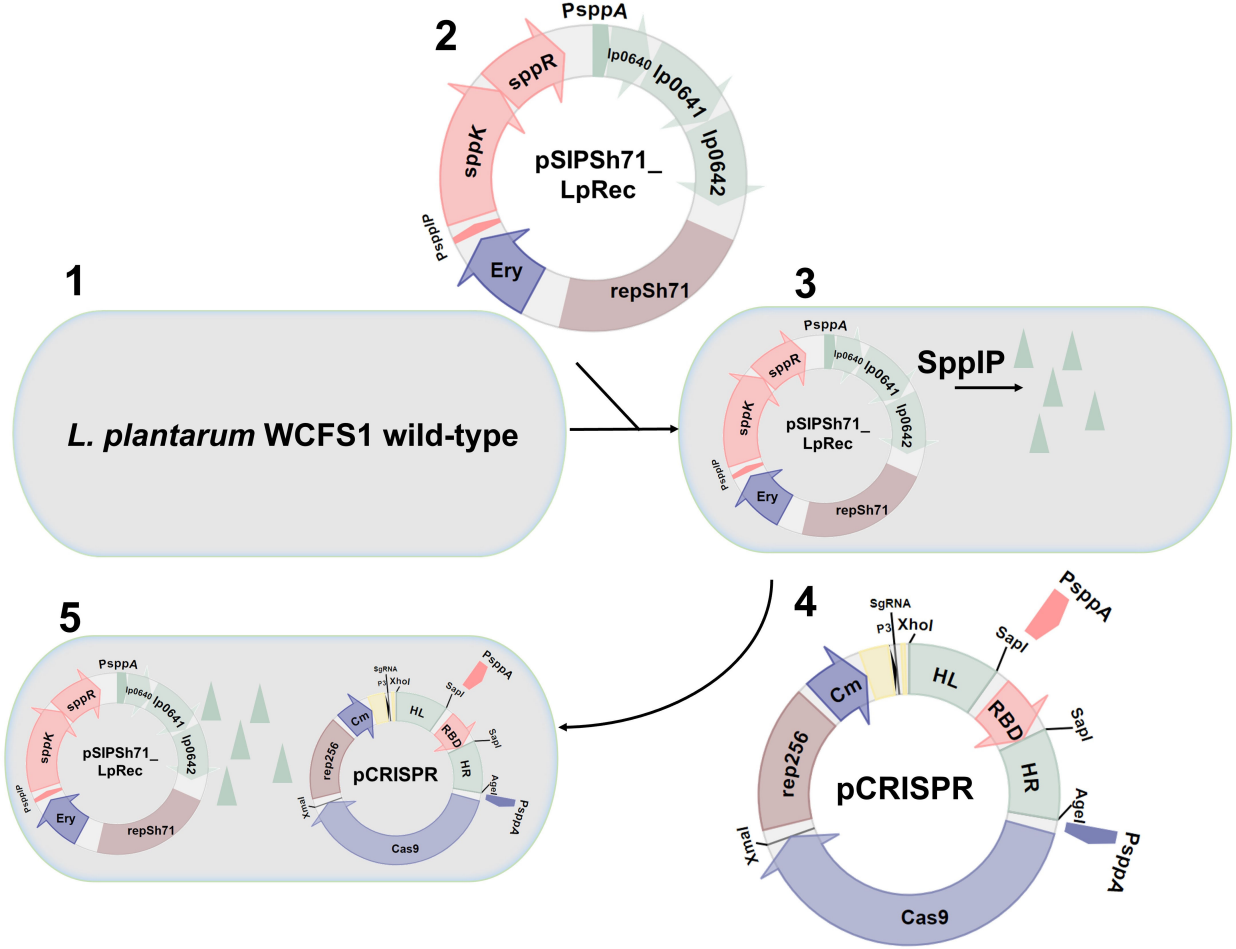
The workflow of the CRISPR-editing system here illustrated for knock-in of RBD with inducible expression. First, the wild-type cells (1) are made competent and transformed (2) with pSIPSh71_LpRec, which also contains *sppK and sppR,* which encode the two-component regulatory system that mediates induction upon the addition of the inducer peptide. Transformants grown in the presence of inducing peptide and thus producing the recombinases (green triangles) are made competent (3) and transformed with a pCRISPR-plasmid (4) to yield the final transformant containing both plasmids (5). The final transformants are plated on MRS agar dishes containing 10 µg/mL Ery, 10 µg/mL Cm, where the HDR occur.

The procedure used to construct knock-in strains consists of five steps, as shown in Fig. 2. Competent *L. plantarum* WCFS1 cells (step 1) were transformed with pSIPSh71-LpRec carrying *lp_0640–42* under control of the inducible P*_sppA_* promoter (step 2). The *L. plantarum* cells harboring pSIPSh71_LpRec were made competent (step 3) and transformed with each of the pCRISPR plasmids (step 4). Recombineering occurred during growth of the transformants on MRS plates containing Ery and Cm in cells harboring both the pSIPSh71_LpRec and a pCRISPR-plasmid (step 5). To assess the effect of the level of recombinase on the knock-in efficiency, we prepared competent cells with various expression levels of these proteins by inducing the bacteria with final concentrations of 0, 0.25, or 25 ng/mL of the inducer pheromone SppIP (see *Material & Methods*).

**Fig 3:**
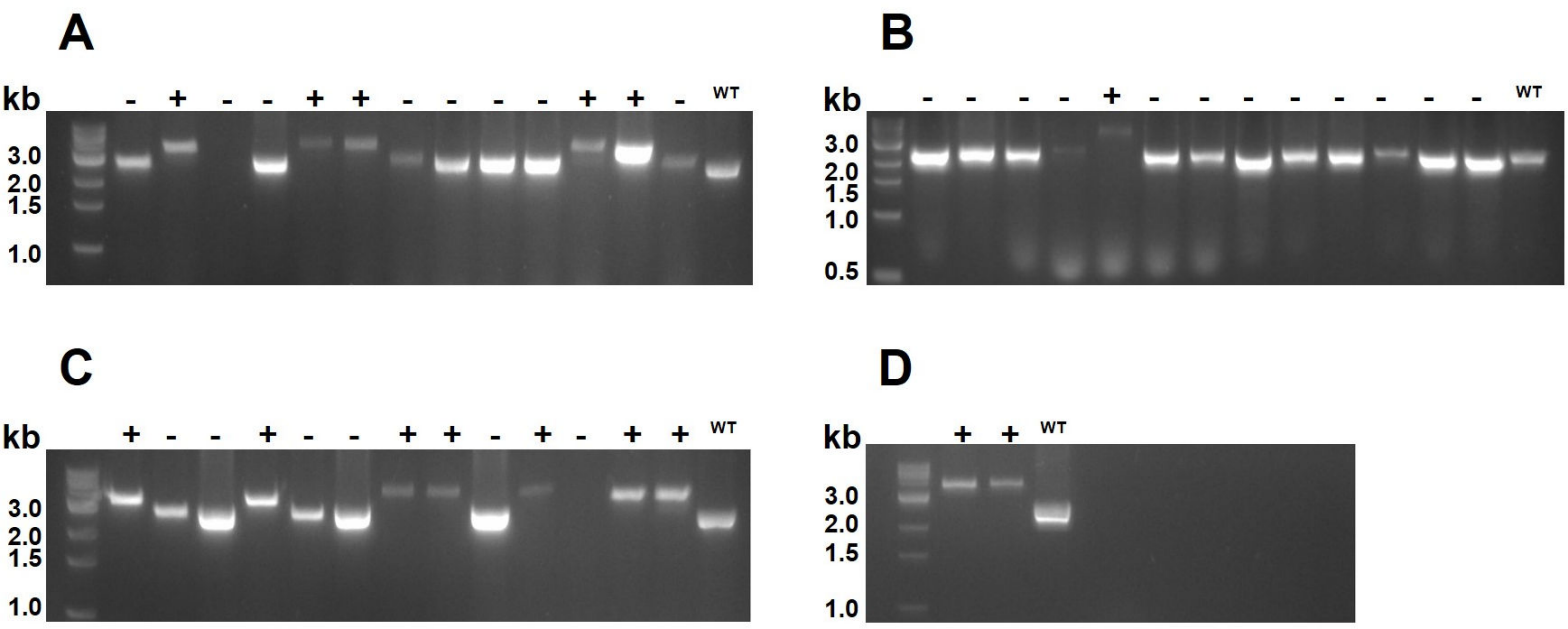
Analysis of a selection of colonies using colony PCR. The PCR products in (**A–D**) mostly correspond in size to the wild-type (2,519 bp) or to inserted P*_sppA_*-mCherry (3,388 bp, **A**), P*_slpA_*-mCherry (3,787 bp; **B**), P*_sppA_*-RBD (3,315 bp; **C**) or P*_sppA_*-Lipo-RBD (3,558 bp; **D**). The wells are marked with either “+” (knock-in colony) or “-“ (WT or not correctly edited colony). Lanes containing the wild-type control PCR product are marked with a “WT” above all four gels. Table 2 shows the total number of colonies screened and the overall knock-in efficiencies.

*L. plantarum* harboring pSIPSh71_LpRec and subjected to varying levels of inducer peptide were made competent and transformed with one of the pCRISPR plasmids for insertion of P*_sppA_*-mCherry, P*_slpA_*-mCherry, P*_sppA_*-RBD, or P*_sppA_*-1261-RBD (Fig. 2, Table 1). Successful recombination was confirmed by colony PCR, where wild-type genomic DNA yields a ~ 2,500-bp band, while the PCR product after correct genomic insertion of P*_sppA_*-mCherry, P*_slpA_*-mCherry, P*_sppA_*-RBD, or P*_sppA_*-Lipo-RBD yields bands of 3,388, 3,787, 3,315, or 3,558 bp, respectively (Fig. 3). In a control experiment, a plasmid encoding the sgRNA and the P*_sppA_*-mCherry expression cassette flanked by the homologous arms, but *not* encoding Cas9, was transformed to the same competent cells. In the absence of the selection pressure exerted by Cas9-cleavage, the control experiment yielded a large number of colonies (>120 colonies per plate). Screening of 30 colonies from three plates for two independent transformations (180 colonies in total) by PCR of the genomic site for insertion showed that none of the screened colonies had undergone recombination (data not shown).

Table 2 shows the total numbers of colonies obtained, all of which were screened, and the overall knock-in efficiencies. The numbers show different transformation efficiencies and different HDR efficacies. As to the latter, the HDR efficacy was positively correlated with the amount of the inducing peptide used during preparation of competent cells for the second transformation step, indicating the pertinence of *lp_0640–42* overexpression. Interestingly, the number of obtained transformants also seemed correlated with the amount of the inducing peptide, whereas there was no correlation between the number of transformants and HDR efficacy. For example, transformation of the largest insert, P*_slpA_*-mCherry (1,291 bp), yielded the most colonies, 72 in total. However, the frequency of correct knock-in was very low (1.3% overall) as the HDR had only occurred in one transformant. Notably, P*_slpA_*-mCherry is the only expression cassette with a constitutive promoter. The HDR efficacy varied between 25% and 100%, for the other expression cassettes. For example, 100% efficacy was obtained for pCRISPR-P*_sppA_*-Lipo-RBD, but in this case, only two transformants were obtained.

**TABLE 2:** Overview of the knock-in efficiency for the four different expression cassettes. The “Induction” column shows the final concentration of SppIP in the culture used to generate competent cells (0, 0.25, or 25 ng/mL SppIP) for the final transformation step (i.e., step 4 in Fig. 2)^*a*^

Knock-in expression cassette	Size of insert (bp)	Amount (µg) of the plasmid transformed	Induction (ng/mL SppIP)	Number of colonies	Correct knock-in	Efficacy (%)
P*_sppA_*-mCherry	890	2.8	0	4	1	25
			0.25	4	2	50
			25	12	5	42
P*_slpA_*-mCherry	1,291		0	12	0	0
		1.6	0.25	25	0	0
			25	35	1	3
P*_sppA_*-RBD	819	2.4	0	0	N/A	N/A
			0.25	5	2	40
			25	10	6	60
P*_sppA_*-Lipo-RBD	1,062	2.2	0	0	N/A	N/A
			0.25	1	1	100
			25	1	1	100

^*a*^ N/A, not applicable.

### Analysis of the knock-in strains

All strains with correct knock-ins were cured for the pSIPSh71_LpRec and pCRISPR plasmids, as described in the *Materials and Methods* section, before proceeding with characterization of the strains.

As mentioned above, we attempted to create knock-ins with both constitutive and inducible expressions of the gene of interest as we assumed that constitutive expression might give problems and low knock-in efficiency (which was indeed observed; Table 2). Activation of the used inducible promoter, P*_sppA_*, depends on the presence of SppK and SppR that sense the inducing peptide and activate the promoter (37). As these genes are not present in the knock-in strains, a helper plasmid (pEV) encoding *sppK* and *sppR,* controlled by the promoter P*_sppIP_*, was transformed into the three knock-in strains with inducible expression, Lp::P*_sppA_*-mCherry, Lp::P*_sppA_*-RBD, and Lp::P*_sppA_*-Lipo-RBD.

**Fig 4:**
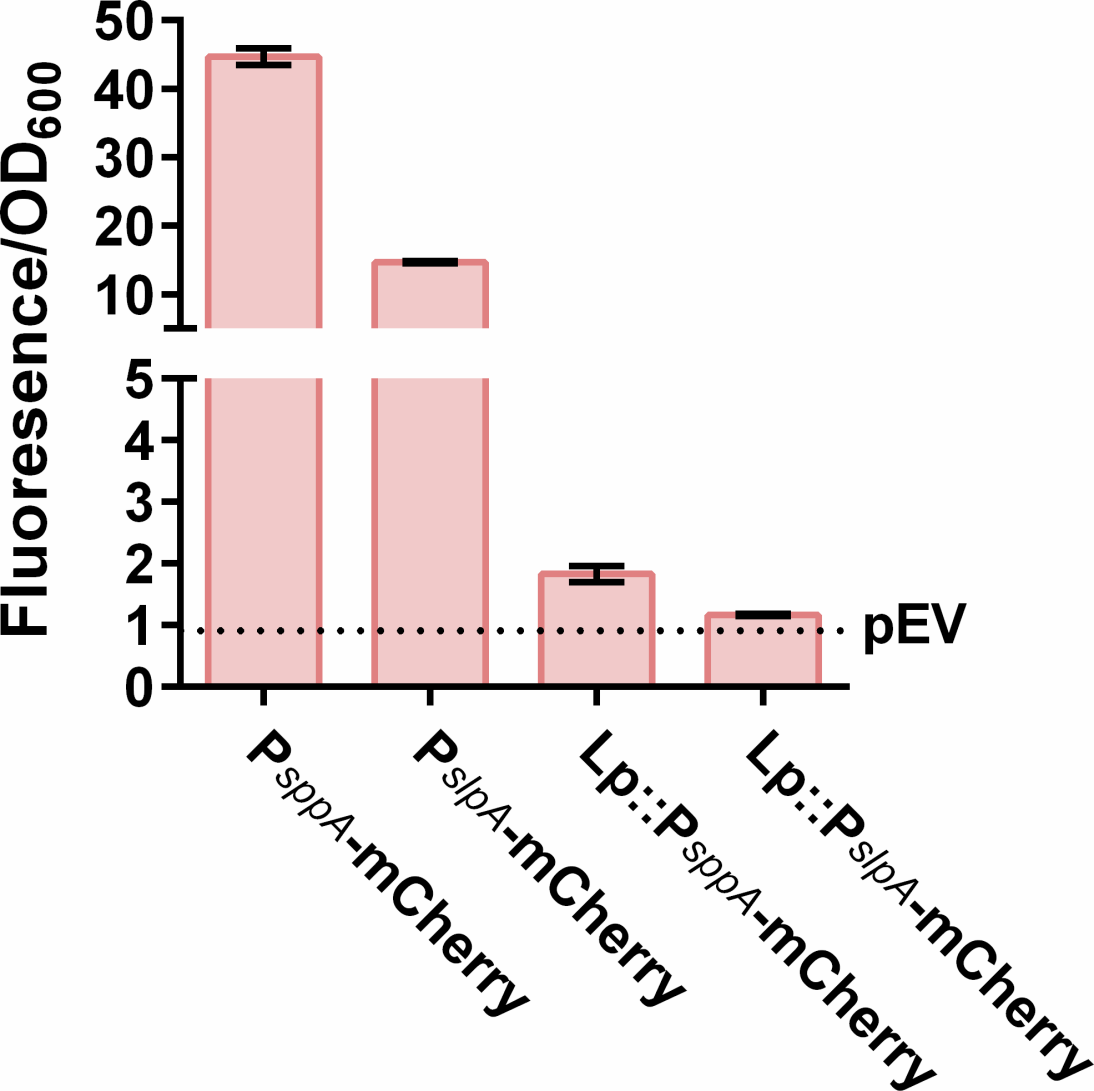
Comparison of the fluorescence signal for strains with plasmid-encoded mCherry and strains carrying (knocked-in) chromosomal mCherry. All strains were diluted from overnight cultures to OD_600_ ~0.15, and strains carrying the P*_sppA_* promoter were induced when the OD_600_ reached ~0.30. Directly after induction of the inducible strains, all strains were transferred to 96-well plates, and continuous measurements were obtained of both the absorbance (OD_600_) and fluorescence (Ex_586_/Em_610_). The figure exhibits the fluorescence/OD_600_ at 3 hours after induction. The horizontal dotted line indicates the fluorescence/OD_600_ of the negative control strain, carrying pEV. The error bars show the standard deviations for three biological replicates.

The construction of mCherry knock-in strains was undertaken to facilitate straightforward and direct comparisons between knock-in strains and those featuring plasmid-based mCherry expression. To analyze the production of mCherry in the knock-in strains, Lp::P*_slpA_*-mCherry and Lp::P*_sppA_*-mCherry, the fluorescence signal was monitored during growth (Fig. 4). For the latter inducible strain, gene expression was induced by addition of the SppIP peptide (25 ng/mL). The fluorescence signal emitted by induced Lp::P*_sppA_*-mCherry showed a twofold increase compared to the signal emitted by Lp::P*_slpA_*-mCherry (*P* < 0.01 in an independent samples *t*-test). While both knock-in strains, in particular Lp::P*_sppA_*-mCherry, show a mCherry signal surpassing the background signal (*P* < 0.01 for both knock-in strains in an independent-samples *t*-test), the fluorescence signals are considerably weaker than those obtained with strains carrying the plasmid-encoded mCherry. Still, these results show that the knocked-in expression cassette is functional.

**Fig 5:**
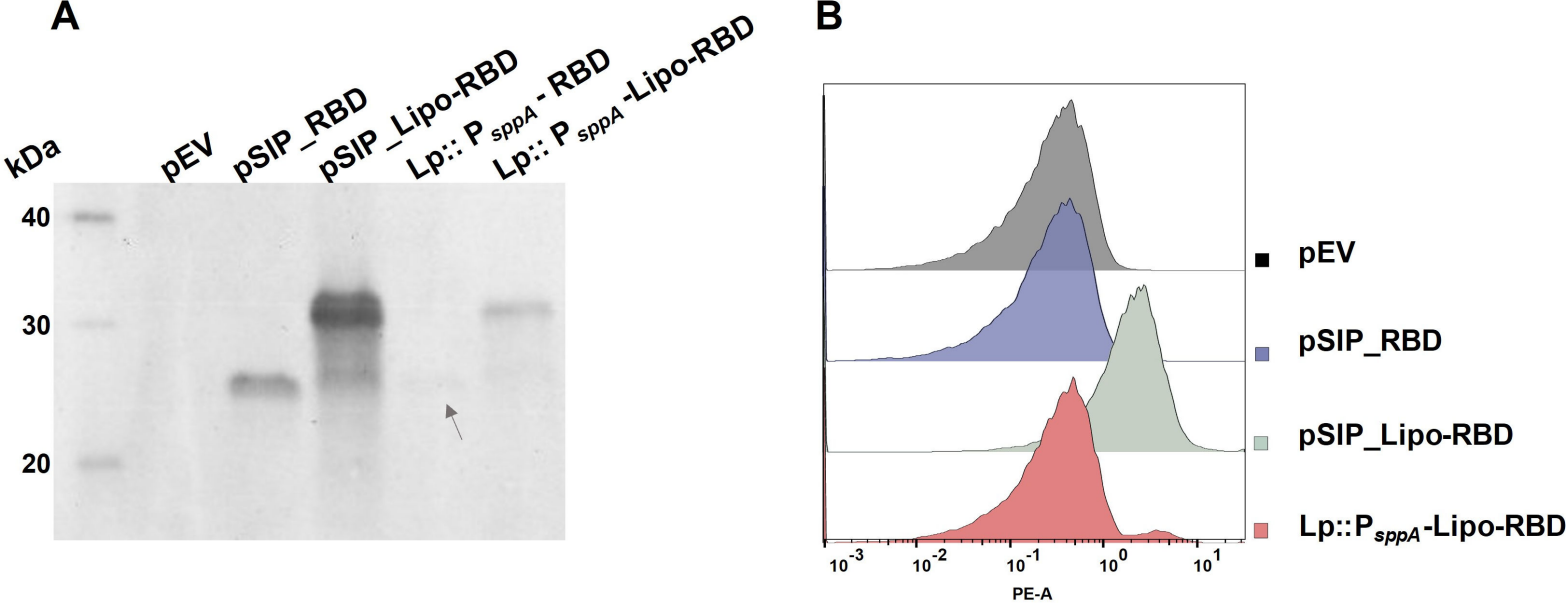
Characterization of knock-in strains containing RBD expression cassettes with Western blotting (**A**) and flow cytometry (**B**). (**A**) Presence of RBD in crude cell-free protein extracts from the various strains analyzed by Western blotting. Lanes: MagicMarker, empty vector (pEV) negative control, plasmid-encoded RBD directed to the cytoplasm (pSIP_RBD; expected size ~25 kDa), plasmid-encoded RBD with the 1,261 lipoanchor (pSIP_Lipo-RBD; ~33 kDa), chromosomally encoded RBD targeted to the cytoplasm (Lp::P*_sppA_*-RBD; ~25 kDa, gray arrow), and chromosomally encoded RBD with the 1,261 lipoanchor (Lp::P*_sppA_*-Lipo-RBD; ~33 kDa). (**B**) Analysis of surface-displayed Lipo-RBD produced either using a plasmid- (pSIP_Lipo-RBD) or chromosomally encoded (Lp::P*_sppA_*-Lipo-RBD) with flow cytometry. pEV and pSIP_RBD (plasmid-encoded intracellular production of RBD) were included as negative controls. A total of 30,000 bacteria were analyzed for each strain.

The fitness of the knock-in strains was comparable to the fitness of *L. plantarum* harboring pEV (Fig. S1). A Western blot was performed to examine the production of the RBD antigen in the knock-in strains. Similar to the mCherry knock-in strains, the analysis revealed notably lower protein levels in the knock-in strains compared to the strains with plasmid-encoded RBD directed to the cytoplasm or to the cell surface (Fig. 5A). The RBD level seemed higher in Lp::P*_sppA_*-Lipo-RBD (surface displayed RBD) compared to Lp::P*_sppA_*-RBD (cytoplasmic RBD).

We also assessed surface display of Lipo-RBD for both the knock-in strain and a strain with plasmid-mediated production of Lipo-RBD. Similarly to the Western blot, the strain with plasmid-encoded Lipo-RBD expression showed a markedly higher amount of antigens on its surface compared to Lp::P*_sppA_*-Lipo-RBD. However, the small peak in the Lp::P*_sppA_*-Lipo-RBD sample corresponds to a fraction of cells that exhibit a shift in the fluorescence, indicating that some of the bacteria have surface-displayed antigens. To ensure that the reason for the detected dual peak was not due to gene instability, a PCR was performed on cells from the same culture as those analyzed with flow cytometry. The PCR showed that P*_sppA_*-Lipo-RBD is still present in the genome (Fig. S2). These cells had been subjected to several subcultivations after the initial verification of expression cassette insertion, and the control experiment in Fig. S2 thus shows that the genomic insertion of P*_sppA_*-Lipo-RBD is stable. Taken together, the results from the Western blotting and flow cytometry show that the knock-in strains indeed produce RBD.

## DISCUSSION

In this study, we developed an inducible two-plasmid system for CRISPR/Cas9-based genome editing of *L. plantarum*. Constructing the plasmids with a cassette-like structure with easily exchangeable components is a step toward the routing implementation of CRISPR/Cas9 genome editing in *L. plantarum*. The system allowed successful knock-ins of four different expression cassettes, ranging from 800 to 1,300 bp, into the genome of *L. plantarum* WCFS1. Although the efficacy varied among expression cassettes to be knocked-in, several of the experiments showed efficacies between 40% and 60%.

One of the plasmids encodes the recombinase operon *lp0640-42*, and our data confirm the importance of the encoded recombinase machinery in facilitating successful knock-ins. For example, 16 colonies in total were obtained when the recombinase protein production was not induced, and only one of these had the expression cassette knocked-in. Apparently, in the absence of the recombinase, the bacterium uses repair pathways other than HDR to survive. Although HDR is considered the preferred repair pathway in prokaryotes, the nonhomologous end joining (NHEJ) pathway is also present in some species (38). NHEJ systems have not been characterized for *L. plantarum* WCFS1, but four predicted proteins share 31%–60% identity with YkoU and eight predicted proteins share 21%–43% identity with YkoV, two proteins known to be involved in NHEJ in *Bacillus subtilis* (39). Since NHEJ is not the preferred repair pathway, rescue would probably occur less frequently if only NHEJ is available. This can explain why induction of recombinase expression correlated with the amount of transformants.

In a previous study, it was shown that the efficacy of gene insertion depends on the length of the homologous arms (18). The knock-in efficiency of a chloramphenicol resistance gene increased when 1400-bp arms were used instead of 950-bp arms. In the present study, we used arms of 1000 bp, based on previous observations regarding CRISPR/Cas9-mediated genome editing, mainly for gene deletions, in *L. plantarum* (23, 25–27). We obtained the best results (i.e., a sufficient amount of transformants and high fraction of successful integrations) with the two shortest expression cassettes (~800–900 bp) compared to the longer ones (>1,000 bp) (Table 2). Longer DNA fragments may require longer homology arms to facilitate efficient recombination, and the HDR may be less efficient compared to shorter fragments due to the increased complexity of aligning and integrating longer sequences. In light of the present results, it is noteworthy that studies with *Pseudomonas syringae* (40) and *L. plantarum* WCFS1 (18) have suggested that recombineering is favored when the chromosomal region removed during recombination is longer than the expression cassette being inserted. Contrasting with these suggestions, we demonstrate the capacity of *L. plantarum* WCFS1 for recombination, even when removing only a minimal part (30 bp) of the chromosome.

Our analyses demonstrated that all four knock-in strains express the inserted target gene. The protein levels were considerably lower in the knock-in strains compared to strains expressing the same protein from plasmid-encoded genes. This is an expected difference, in accordance with previous observations made by X. Song *et al*. (23), who knocked-in *gfp* under control of the strong P_23_ promoter into the genome of *L. casei*. Similar to P_23_, the promoters used in our study, P*_sppA_* and P*_slpA_*, are known as strong promoters. Variations in the gene dosage can partly explain the relatively poor protein production levels in the knock-in strains. It is known that pSIP401 derivatives used in the present study have a copy number of approximately 6 in *L. plantarum* (8, 9).

The site of chromosomal insertion may be another factor determining expression levels. This effect, which may relate to unwinding of the DNA to enable polymerase binding, is well-known in eukaryotic cells and has also been observed in bacteria (41). A study by J. A. Bryant *et al*. (42) showed that the protein production encoded by a lac-*gfp* expression cassette inserted in the *E. coli* chromosome varied up to ~300-fold depending on the chromosomal location. They also observed cases of complete silencing of the expression. Further analyses showed that the number of *gfp*-associated RNA polymerases varied based on the chromosomal locus, which also correlated with GFP fluorescence levels. To our knowledge, the chromosomal organization of *L. plantarum* WCFS1 has not yet been characterized in terms of nucleoid organization. We inserted the expression cassettes between two genes transcribed in opposite directions to avoid possible negative interference with transcription of native genes, but otherwise, the choice of the site was more or less random. It is conceivable that the insertion site may be part of a poorly transcribed region of the *L. plantarum* chromosome. Regions known to be highly expressed, such as regions encoding ribosomal proteins (43), could be interesting targets in future experiments. It is interesting to note that the editing efficiency too could be enhanced by targeting chromosomal regions more abundantly associated with RNA polymerases. S. Gong *et al*. (44) demonstrated that unwinding of DNA promotes Cas9 cleavage of dsDNA. The association of DNA with RNA polymerases leads to unwinding of the DNA, enabling R-loop formation between the sgRNA/Cas9 complex and the target sequence. On a side note, in future strain engineering, the regulatory *sppKR* cassette, which was supplied on the pEV helper plasmid in the present proof-of-concept study, should be integrated into the host strain genome. Similar regulatory elements from *L. plantarum* itself are available (45).

Regarding the RBD-knock-in strains, it is interesting to note that the surface-anchored protein seems to be produced more efficiently than the nonsecreted protein, regardless of whether the expression cassette is plasmid-based or integrated into the genome. This difference may be due to intracellular protease activity targeting the RBD, while the secreted Lipo-RBD may be partially protected through interactions with components of the secretion machinery. However, these differences may also be due to differences in transcription levels or mRNA stability.

To the best of our knowledge, there are no examples of successful CRISPR/Cas9-mediated insertion of a gene encoding surface-displayed protein into the genome of a gram-positive bacterium. While the flow cytometry data confirm low protein expression levels, Lipo-RBD does appear on the cell surface of the knock-in strain. Data for the control strain producing plasmid-encoded Lipo-RBD clearly show anchoring of the RBD antigen in an exposed (i.e., immunologically detectable) position on the lactobacillal surface. Considering the potential for vaccine development, it is encouraging that RBD can be expressed and surface-displayed in *L. plantarum*.

In conclusion, we have developed an easy-to-use two-plasmid system that allows insertion of various expression cassettes into the chromosome of *L. plantarum*. The knock-in plasmid is designed to facilitate easy exchange of the target fragment and the homologous arms. The combination of a relatively low number of colonies after the second transformation and, in most cases, a high knock-in frequency makes it easy and fast to identify correctly engineered strains. The protein production from the knock-ins was substantially decreased compared to plasmid-mediated expression of the same proteins, and further work is needed to see if and how protein production levels can be improved. Importantly, this study demonstrates the first example of the production and correct targeting of a heterologous surface-anchored antigen in a CRISPR-Cas edited gram-positive bacterium, indicating that the developed system may have value for the construction of lactobacilli-based vaccine delivery vehicles.

## Data Availability

The raw data supporting the conclusions of this manuscript have been made available at the figshare depository: https://doi.org/10.6084/m9.figshare.28194110.v1.

## SUPPLEMENTAL MATERIAL

Supplemental material spectrum.02025-24-s0001.docx Fig. S1 and S2; Table S1.

ASM does not own the copyrights to Supplemental Material that may be linked to, or accessed through, an article. The authors have granted ASM a non-exclusive, world-wide license to publish the Supplemental Material files. Please contact the corresponding author directly for reuse.
