# Supplementary material for: CRISPR/Cas9-mediated genomic insertion of functional genes into Lactiplantibacillus plantarum WCFS1
Source: Microbiol Spectr. 2025 Jan 16;13(2):e02025-24. doi: 10.1128/spectrum.02025-24 (PMC11792511; doi:10.1128/spectrum.02025-24)
Supplement: Supplemental material — Fig. S1 and S2; Table S1. [file spectrum.02025-24-s0001.docx]

**CRISPR/Cas9 mediated genomic insertion of functional genes into Lactiplantibacillus plantarum WCFS1**

Kamilla Wiull^1*^, Lisa K. Haugen^1^, Vincent G.H. Eijsink^1^, Geir Mathiesen^1*^

^1^ Faculty of Chemistry, Biotechnology and Food Science, NMBU - Norwegian University of Life Sciences, Ås, Norway

**Supplementary Material**

**

**

**Figure S1.** Fitness of the RBD knock-in strains. Cultures of each strain were transferred to a 96-well plate after induction with 25 ng/ml SppIP (time = 0 h). The OD_620_ was measured continuously, every hour for 20 hours. Three biological replicates and two technical replicates were included for all three strains. The datapoints show the mean of the three replicates at every datapoint and the error bars shows the standard deviation between the replicates.

**
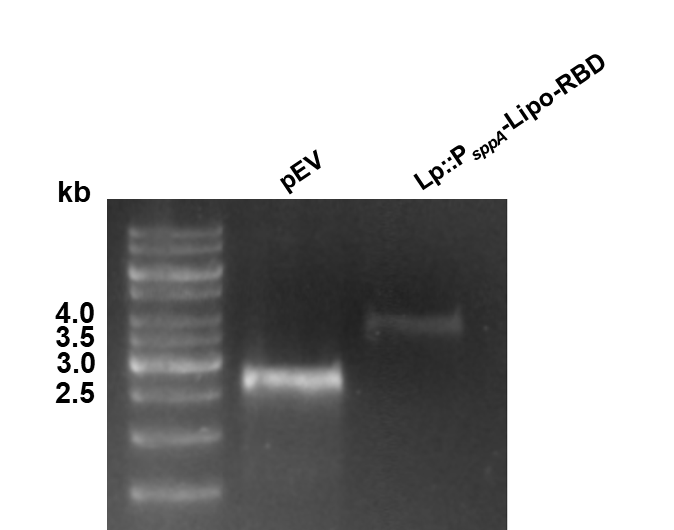
**

**Figure S2.** Verification of knock-in stability of Lp::P*_sppA_*-Lipo-RBD. The stability was assessed in the cells used for flow cytometry analysis by PCR amplification of the genomic region targeted for insertion. Cells containing pEV (wild-type genome sequence) were used as a negative control. The expected sizes of the fragments are 2.5 kb without insertion (pEV) and 3.6 kb with insertion (Lp::P*_sppA_*-Lipo-RBD)

**Table S1.** Primers used in this study. Restriction sites appear in italics and are underlined.

| **Name** | **Sequence** | **Description** |
| --- | --- | --- |
| Phospho-sgRNA_R | TATAGTTATTATACCAGGGGGACAGTGC | Phosporylated primer; binds any pSgRNA-plasmid. |
| SgRNA_KI1_F | **ATAAACGACTTCGG**  **TGGAAT**GTTTAAGA  GCTATGCTGGAAACAG | Forward primer for inserting the sgRNA (bold) between *lp_2071* and *lp_2074*, called “KI”. |
| Cas9NcoI_F | AGTATGATTC*CCATGG*ATAAGAAATACTCAATAGGCTT | Primer pair for amplification of Cas9 from the pCas9 plasmid for insertion into pSIP_403. |
| pCasR | ACCGAATTC*CTCGAG*TCAGTCACCTCCTAGCT |  |
| Sg-Cas9_R | CGCCTTCGAA*CCCGGG*TCAGTCACCTCCTAGCTGA | Primer pair for amplification of P*_sppA_*-Cas9 from the pCas9 plasmid. |
| SgRNAKI-1-HA_cas9_F | CCTCCAGTAA*CTCGAG*GATCTACCGGTTTAATTTGAAAATTGATATTAGC |  |
| pSgKI1-HL_XhoI_F | CCTCCAGTAA*CTCGAG*CTACAAGATCCACCAACT | Primer pair for amplification of the region upstream of the sgRNA target site, called homologous arm left (HL). The reverse primer carries a tail homologous to RBD. |
| HL_2071-2074_SapI_rev | *GCTCTTC*TTATGGGCTAATAACAAGC |  |
| H-2071-2074-R_fwd | CACAGCGTCCATAGAAGCTT*GCTCTTC*ATAATCCTACTTGGTTAGACTG | Primer pair for amplification of the region downstream of the single guide RNA target site, called homologous arm right (HR). The forward primer contains a tail homologous to RBD. |
| pSgKI1-HR_AgeI_R | ATATGGGCCC*ACCGGT*ACGAGCCAGACAGTTTTAAGT |  |
| RBD_F | TAGGAGTATGATT*CATATG*CCAAACATCACGAACTT | InFusion primers for amplification of RBD from pUC57_DC_NTD_RBD. |
| RBD_R | CTGTAATTTG*AAGCTT*CTATGGACGCTGTGGGGT |  |
| 1261_RBD_F | GATTGCGGCG*GTCGAC*CCAAACATCACGAACTTGTG | InFusion primers for amplification of RBD from pUC57_DC_NTD_RBD for insertion of RBD into pLp_1261AgE6-DC. |
| 1261_RBD_R | CTGTAATTTG*AAGCTT*CTATGGACGCTGTGGGGTTGAATGGTATGATGGATAAAAGCCTGAACCGCAAACTGTGGCT |  |
| Psppa-RBD_fwd | GCTTGTTATTAGCCCATAA*GAAGAGC*ACCGGTGGGCCCATATTAAC | Primers used to amplify P*_sppA_*-RBD from pSIP_RBD. The forward primer has a tail homologous to HL, while the reverse primer has a tail homologous to HR. |
| KI1_HR_RBD_R | AGTCTAACCAAGTAGGATTAT*GAAGAGC*AAGCTTCTATGGACGCTG |  |
| HL_slpA-RBD _fwd | TGCTTGTTATTAGCCCATAA*GAAGAGC*AGATCTATAAAGTTGTTTGATAAATGC | Primer for amplification of P*_slpA_*-mCherry. |
| KI1_HR_mCherry_R | AGTCTAACCAAGTAGGATTAT*GAAGAGC*GAAGCTTTTATTTGTAAAGCTCATC | Reverse primer for amplification of either P*_sppA_*-mCherry or P*_slpA_*-mCherry. |
| SgRNA-LpRec_F | CCTCCAGTAACTCGAG*ACCGGT*GGGCCCATATTA | Primer pair for amplification of P*_sppA_*, with a tail homologous to *lp0640-42*. |
| SppA-LpRec_R | TTTCGGAGTA*CATATG*AATCATACTCCTATATATTATTTTATAAAGCA |  |
| SppA-LpRec_F | GAGTATGATT*CATATG*TACTCCGAAACAGTCACTAAGA | Primer pair for amplification of the *lp_0640-42* operon from the *L. plantarum* genome, with a tail homologous to P*_sppA_*. |
| SgRNA-LpRec_R | TCGAA*CCCGGGG*TACCTCAATCTATGAGTAAGTCGTCTG |  |
| 2071-2074_SekF | ATGAAAACCATGAGTCTTGT | Primers used for verification of engineered *L. plantarum strains*, binding to the *L. plantarum* genome outside of the homologous arms. |
| 2071-2074_SekR | TGATGAAGCTAAGGCTGAT |  |
